# The Role of Self-Interest in Deliberation: A Theory of Deliberative Capital

**DOI:** 10.1177/0032321720981491

**Published:** 2021-01-20

**Authors:** Afsoun Afsahi

**Affiliations:** Department of Political Science, University of Amsterdam, Amsterdam, The Netherlands

**Keywords:** deliberative capital, social capital, self-interest, deliberative democracy, investments, divestments, facilitation

## Abstract

How do successful deliberations unfold? What happens when they unravel? In this article, I propose that we think of the dynamics of participant engagement within deliberation as series of self-interested and reciprocal investments in and divestments from *deliberative capital*. This article has three parts. First, I draw on the literatures on deliberative democracy and social capital to outline a theory of deliberative capital. I highlight the important role self-interest plays in the process of those initial investments – instances of engagement in positive deliberative behaviours. Second, drawing from my experience as a facilitator, I give an account of the particular indicators of investments and divestments that we might expect to see in a given deliberative engagement. Third, I briefly outline two innovative facilitation techniques that can be utilized at the beginning or during a deliberative process that trigger self-interest, which incentivizes investments and discourages divestments.

Over the past three decades, deliberative democracy has become ubiquitous. Deliberative democracy ‘is premised on the assumption that under the right conditions, people are able and willing to communicate with each other in an open manner and offer reasons and explanations for their positions’ and make decisions together (Afsahi, 2020a: 3). Both democratic theorists and empirical political scientists have looked to deliberation with its promises of ‘better-informed and more engaged [citizenry] [. . .] aware of their own interests and tolerant of the values of others’ (Afsahi, 2020b: 1) as a panacea to problems of legitimacy and accountability. Experiments in deliberative democracy have ‘refute[d] many of the more pessimistic claims about the citizenry’s ability to make sound judgments’ and mini-publics carried out under conditions of current and past conflict in ‘Colombia, Belgium, Northern Ireland, and Bosnia [have shown] that properly structured deliberation can promote recognition, understanding, and learning’ (Dryzek et al., 2019: 1145). In fact, between January 2010 and January 2020, more than 850 participatory processes around the world brought individuals together to deliberate and make decisions together (Fung and Warren, 2020). Considering the increasing use of deliberative and participatory practices in consultation and governance processes, this article takes as its starting point the value of small-scale deliberation as a useful and even necessary tool within democratic systems (for a discussion on this, see Beauvais and Warren, 2019; Warren, 2017). Instead of challenging the basic presumptions of deliberative democratic theorists and practitioners, I focus on the dynamics within such small-scale deliberations and ask: how do successful deliberations unfold? What happens when deliberations unravel? These are not novel questions. Many scholars have attempted to crack the ‘black box’ of deliberation (Mendelberg et al., 2014; also see Jaramillo and Steiner, 2014; Mansbridge et al., 2006; Steenbergen et al., 2003; Steiner et al., 2004). My response, however, is novel. I propose that we think of the dynamics of participant engagement within deliberation as series of self-interested and reciprocal investments in and divestments from *deliberative capital*.

This article has three sections. First, I draw on the literature on deliberative democracy and social capital to outline a theory of deliberative capital. I posit investments as instances of engagement in positive deliberative behaviours and reframe engagement in negative deliberative behaviours as instances of divestments. I highlight the important role self-interest plays in the process of those initial investments in a deliberative engagement. Self-interest rather than a generalized commitment to common interest or general good establishes the possibility of initial investments and the subsequent virtuous cycles.

This new focus on self-interest explains how we may, and often do, overcome the problem of *pre-commitment* within deliberative democratic practices. Successful deliberations are in need of participants who show up with some degree of *pre-commitment* to the basic norms of deliberation such as justification, respect, listening and reciprocity. However, this may not be the case and we have no way of assuring that we have the *right kind* of participants – nor can we see our deliberation as *inclusive* if we only have individuals who are already committed to such norms. This problem is further complicated by theorists and practitioners who argue that commitment to these norms is an *outcome* of deliberation – something that participants learn and become better at as a result of participating (for a brief discussion, see Beauvais, 2020). So how do we get around this causality dilemma? I argue that we do so by recognizing the role that self-interest plays in kickstarting reciprocal investments.

Second, I go into detail about what constitutes an investment or a divestment during deliberation. Drawing from the literature on deliberative democracy and my experience as a facilitator, I give an account of the particular indicators of investments and divestments that we might expect to see in a given deliberative engagement.^1^ Finally, I briefly outline two innovative facilitation techniques that can be utilized at the beginning or during a deliberative process to incentivize investments and discourage divestments – or to get deliberation back on track. The first is simulated representation, which gets participants to switch places by learning, presenting and defending each other’s views for a portion of deliberation. The second facilitation technique is that of deliberative worth exercises, which asks participants to keep track of each other’s behaviours and rate each other based on their respective investments and divestments in order to pick the best interlocutors of each round of deliberation.

## Deliberative Capital

What is deliberative capital?^2^ Deliberative capital can be conceptualized as the by-product of the investments – instances of positive deliberative behaviours – made by participants during the course of the deliberative process. Investments – which can include behaviours such as explaining one’s reasons, waiting for one’s turn or offering a compromising proposal – make it more likely for others to do the same and as such increase the overall deliberative capital in a particular deliberation. This reciprocal process in turn facilitates a better and easier dialogue process for all participants. This establishes the value of deliberative capital as a *means* to an end. It is defined and identified by its productive function: producing better and easier conversations. Without a sufficient degree of deliberative capital, good deliberation – one that is open, respectful and constructive – will not come about.

From this description, it becomes clear that deliberative capital can also be easily threatened when these investments are replaced by divestments. These behaviours – which can include expressing marginalizing comments, cutting others off or rejecting compromising proposals without considering them – can derail the process of deliberation by increasing the chances that other participants behave similarly and reciprocally.

As noted above, I draw on the existing literature to compile the list of investments and divestments. However, I argue that we cannot think of these investments and divestments as functionally separate from one another. It is in the framing of these investments and divestments as reciprocal cycles contributing to or detracting from deliberative capital that my contribution lies. I argue that such a framing is important because it allows us to see a deliberative engagement as an organic whole, which is affected by the particular actions of the participants – whether positive or negative. Investments beget investments and divestments make future divestments more likely. This framing highlights the necessity of not only finding ways to encourage investments but cutting the cycles of divestments as soon as possible within deliberation.

### (Social) Capital

Recognizing the reciprocal relationship between investments and divestments underscores the value of the concept of *capital*, which incorporates the process of investing with the expectation of future returns that can occur during a deliberation. The term *capital* has a long history and many uses. In its traditional usage in economics, capital is an already produced good that can be used in the production of more goods and services and is, thus, one of the factors of production alongside land, labour and entrepreneurship (Hicks, 1971: 272). Used more broadly, the term *capital* encompasses other forms of investments and assets: human, academic and cultural, to name a few.^3^

Deliberative capital, as described above, is a form of *relational* capital – one which exists in the bonds between interlocutors within the process of deliberation. While this interpretation or framework of understanding deliberation dynamics as investments in and divestments from deliberative capital is original, it draws considerably from work already done in deliberative democratic theory looking at processes of deliberation in small-scale deliberative engagements as well as the literature on social capital.^4^

Social capital is a collection of ‘actual or potential resources’ – a by-product of the associations between individuals within ‘a durable network of more or less institutionalized relationships of mutual acquaintance or recognition’ (Bourdieu, 1986: 248). These resources broadly include ‘the exchange of favors, the maintenance of group norms, the presence of trust, and the exercise of sanctions [which are] available to members of social groups’ (Villalonga-Olives and Kawachi, 2017: 105). These resources ease the activities of individuals within these networks (see Coleman, 1987, 1988) – particularly those which require ‘coordination and cooperation for mutual benefit’ (Putnam, 1995: 67). Reciprocal investments in social capital can make actions within a particular social structure easier, more cooperative and trusting by reducing the costs associated with social interactions (see Fukuyama, 2003; Lins et al., 2017).

Whether or not (sufficient) social capital exists within a particular structure or not depends on the particular ‘features of [a] social organization such as networks, norms, and social trust that [can] facilitate coordination and cooperation for mutual benefit’ (Putnam, 1995: 67). Initial investments in social capital require a basic degree of trust – or ‘the expectation that another person (or institution) will perform actions that are beneficial, or at least not detrimental, to us regardless of our capacity to monitor those actions’ (Sapienza and Zingales, 2012: 124). This general presumption of goodwill makes it possible for individuals to make initial investments. Subsequent investments in social capital come to rest on general social norms that enforce reciprocity, often by making investing the *socially desirable* action.

Reciprocity within social relations ‘serves to reconcile self-interest and solidarity’ (Putnam et al., 1993: 172). For example, as a result of reciprocity within social capital, it becomes fruitful and advantageous for me to honour my agreement with the neighbour down the street, not only because I would want the same neighbour to do the same for me but that I would want to avoid the negative costs associated with non-compliance if word gets around the neighbourhood that I do not honour my agreements. The likely outcome of such non-compliance is that I will find myself repudiated and ostracized from my community. Therefore, the norm of reciprocity ‘reduce[s] incentives to defect, reduce uncertainty, and provide models for future cooperation’ (p. 178). My compliance becomes a matter of self-interest and community interest – it benefits me and those around me.

Creation and, the sometimes inevitable, destruction of social capital are ‘marked by virtuous and vicious circles’ (Putnam et al., 1993: 170) which are ‘self-reinforcing and cumulative’ (p. 177). Virtuous circles are recognizable by ‘high levels of cooperation, trust, reciprocity, civil engagement, and collective well-being’, whereas ‘[d]efection, distrust, shirking, exploitation, isolation, disorder, and stagnation intensify one another in a suffocating miasma of vicious circles’ (p. 177). One can easily imagine how respective actions and events can reinforce and strengthen one another in an ongoing feedback loop.^5^

### From Social Capital to Deliberative Capital

Deliberative capital is analytically akin to social capital. Deliberative capital too is an accumulation of resources – a by-product of investments made by individuals within a deliberation. This deliberation – whether it takes place during a few hours or over the course of months – constitutes a more or less consistent web of associations between interlocutors who share the experience with one another.

Deliberative capital as a resource works as follows: it eases the exchange of ideas between participants within deliberation; it serves as an impetus for maintaining and reinforcing norms of deliberation; it assures participants that there is something of value for them through their participation; and, finally, it precipitates the implementation of deterrents in the event of divestments by some participants. As such, we can identify a deliberative setting as having ample deliberative capital when participants find it easier to engage with one another in respectful, reflective and constructive ways. Reciprocal investments in deliberative capital reduce the costs associated with investments by turning those investments into the norm of deliberation. Indeed, they increase the costs of behaving otherwise – by divesting.

Whether or not a particular deliberative engagement is able to produce ample deliberative capital depends on a number of factors. Most importantly, however, those early investments by participants require an elementary measure of trust and willingness on the part of the participants to take that extra step at the start and invest in deliberative capital. This initial phase of deliberation is the most precarious – it is the stage where the norms of deliberation are being established. The concept of deliberative capital is particularly useful at capturing and responding to this precariousness, which arises from what I call the *problem of pre-commitment* within deliberative democratic theory and practice.

Theoretically, deliberation requires participants to show up to the table with, at least, a degree of *pre-commitment* to the desired norms of deliberation. In other words, deliberative democracy requires participants to come to the table willing to explain their positions, respect each other, listen to one another, and to take in and respond to one another. Therefore, we need, and often optimistically expect, individuals to explain their positions by expanding either on their reasons or feelings. We see it as critical for participants to similarly be willing to respect each other, listen to one another, and to take in and respond to other interlocutors. Finally, we require deliberators to be open to a degree of give-and-take or constructive dialogue.

However, this might not always be the case. While I might not come to the table set on derailing the deliberative process, I might not be particularly *pre-committed* to the norms of justification, respect and equality.^6^ In fact, commitment to these norms is something that we expect participants to learn and internalize as a result of and during the course of deliberation. This is the *problem of pre-commitment*.

The problem of pre-commitment is an inherited one. One need only to look to Amy Gutmann and Dennis Thompson’s *Democracy and Disagreement* and the notion of ‘conditions of deliberation’ to locate it. They identify reciprocity as one of the conditions of deliberation. Gutmann and Thompson do not provide ‘[t]he precise content of reciprocity’ but argue that ‘[i]t can be seen in the difference between acting in one’s self-interest [. . .] and acting fairly’ (Gutmann and Thompson, 1996: 2). They further identify ‘mutual respect’ as an obligation in cases where disagreement is deliberative (i.e. legalizing abortion) (p. 3). Mutual respect for Gutmann and Thompson requires ‘a favorable attitude toward, and constructive interaction with, the persons with whom one disagrees’ (p. 79). However, it is not clear why or how we would expect individuals to be pre-committed to these key norms of reciprocity and mutual respect (Dryzek, 2005; O’Flynn, 2007). And without a guarantee that deliberation will be guided by these principles of mutual respect and reciprocity, ‘speakers cannot expect deliberation to be the solution’ (Beauvais, 2020: 5) to problems of legitimacy and accountability or to be able to deliver on any of the desired outcomes commonly associated with deliberation.

James Bohman drawing on Aristotle succinctly summarizes this problem: ‘When water chokes, what is one to wash it down with?’ (Aristotle, 1990: Book VII.2, 1146a; Bohman, 2000). When deliberation itself is marred by lack of reciprocity and mutual respect, more deliberation hardly seems to be the vehicle by which we can achieve more reciprocity and mutual respect. Edana Beauvais, in her assessment of this problem, points to mini-publics as sites that can ‘neutralize inequalities in [. . .] institutional settings’ (Beauvais, 2020: 7) and bring about mutual respect and reciprocity. Mini-publics have indeed shown themselves to be capable of doing so.

But how? Since we cannot guarantee that participants will come to the table already committed to these norms necessary for good deliberation, what assurance do we have that deliberative processes can carry out successfully? What is the particular mechanism at work that prompts individuals to *invest* and establish reciprocity as the norm? Once again, the analytical connection between social capital and deliberative capital is illuminating. Initial investments in deliberative capital come about through a process of self-interest incentivizing investments with the hope of future returns from other participants. As Mark Warren argues, ‘good manners do not [. . .] rely on altruism, since individuals rarely get their way through rudeness, while they do well through cooperation’ (Warren, 2006: 175). In other words, we first invest because we want others to do the same. Our investment, in turn, fulfils the expectation of others and acts as a confirmation that they were not wrong to trust in the process.^7^

These initial investments subsequently create an obligation on the part of other participants to behave similarly by continuing the cycle of investments. Their reciprocal investments, fostered in equal parts by concern for self-interest and the pressure of norm compliance, further establish and strengthen deliberative norms. The reinforcement of deliberative norms, then, incentivizes further investments as participants come to realize that their self-interest will not be fulfilled by straying too far from the deliberative norms that others adhere to and risk being penalized by other participants. Investments, when reciprocated, increase the deliberative capital within a deliberation. The more participants invest, the more it becomes a norm and an expectation that they will do so. In other words, the concern for self-interest gets transformed into the norm of reciprocity.^8^

I am far from suggesting that at the outset of deliberation, none of the participants value the norms of deliberation. Instead, my argument is that for good deliberation to occur, we do not necessarily need to rely on participants being pre-committed to these norms. We do not need ideal deliberative citizens, only good ones who are guided by their self-interest to invest in deliberative capital. However, it is important to remember that the flipside can also be possible within deliberation. Divestments can too be guided by self-interest. Participants – commonly volunteers – in a deliberation can be often driven by desire to influence and convince others; in other words: to win the argument. This desire can push participants to divest from deliberative capital particularly if the opportunity presents itself. The absence of a good structure and skilled facilitation can incentivize divestments. If one thinks that she can get away with divestments in order to win the argument, she may very well try it. This is why it is crucial for us to take steps and think through techniques and measures that could further incentivize early investments by reducing the risks and increasing the pay-offs; or to find ways to get participants back on track to invest when they, often inevitably, stray too far and divest.

Despite many analytical similarities, social capital and deliberative capital differ from one another in one crucial aspect: the extent to which the free-rider problem becomes a problem in the process (for a discussion on the free-rider problem, see Levi, 1996; Schmid, 2000). In the case of deliberative capital, the free-rider problem is less likely because the structure is more closed and the interaction cycles are finite. The likelihood that your divestment goes unnoticed or that you can benefit from cycles of investments by others without any investments of your own is lowered. Moreover, the presence of facilitators whose interventions often remind participants of the norms of deliberation incentivize investments in a more direct way than is possible in the case of social capital.

The framework of deliberative capital has three particular advantages. First, the framework of deliberative capital, with its focus on self-interest as a mechanism that pushes participants to invest, demonstrates how deliberations in mini-publics can meet or foster the possibility of meeting the norm of reciprocity. Moreover, while deliberative capital has the most force within the scope and span of a particular deliberative moment, the possibility of it facilitating the development of norms of mutual respect and relational equality – which are required for democratic life – is not precluded.

Second, this framework both complements and addresses some of the gaps in the increasingly utilized Discourse Quality Index (DQI; Steenbergen et al., 2003) and Deliberative Transformative Moments (DTM; Jaramillo and Steiner, 2014) as well as the inductive study of deliberation (Mansbridge et al., 2006) and those concerned with the type and frequency of disagreements within deliberative processes (Stromer-Galley, 2007; Stromer-Galley et al., 2015; Stromer-Galley and Muhlberger, 2009).^9^ Much like DTM, the framework of deliberative capital is interested in mapping the ‘deliberative ups and downs of a group discussion’ (Jaramillo and Steiner, 2014: 2). However, its focus is different. The framework of deliberative capital reinterprets the norm of reciprocity within deliberation by highlighting the important role that self-interest plays as a mechanism fostering adherence to the norm. Investments – engagement in positive deliberative behaviours – are made with the expectation of future returns. Conversely, just as investments are reciprocated, so are divestments. This elucidates and emphasizes the snowballing effect that happens during the course of deliberation, which can elevate the discussion or derail it.

Third, the concept of deliberative capital is more functional and lends itself more easily to empirical investigations than a more ambiguous notion like *deliberative experience* – which similarly aims to capture the overall atmosphere and process of the deliberation. The concept of deliberative experience is not clearly defined and yet is omnipresent within the literature of deliberative democracy (e.g. Gastil et al., 2008; Hickerson and Gastil, 2008; Hough and Park, 2002; Knobloch and Gastil, 2015; Mutz, 2008; Parkinson, 2006). It refers to the overall experience, positive or negative, of participants within deliberation.

The problem with the concept of deliberative *experience* (whether positive or negative) is its ambiguity and impreciseness. Deliberative experience has come to refer to a wide range of outcomes from learning and problem resolution to personal satisfaction, political efficacy and mutual tolerance. This vagueness means that scholars are often talking past one another. It also means that we have a harder time quantifying experience – by having different measures in mind, we can focus on and find varying results. However, by tallying up investments and divestments, we catch a glimpse of the overall deliberative capital and can observe the cycles of investments and divestments – when the investments and divestments are clearly returned and reciprocated within a deliberation (for an empirical study operationalizing these indicators, see Afsahi, 2020b).

Finally, the framework of deliberative capital vindicates the role played by facilitators within a deliberation. Facilitators ease the process of investments by, first, ensuring basic desired structural conditions such as inclusivity, openness, representativeness and equality, and, second, more importantly, by creating a sense of trust in the participants that there is something of value for them in the next hour, or weeks, or months to come. Moreover, with more careful attention to practices and exercises that can incentivize investments, facilitators play a key role in transforming cycles of divestments to cycles of investments (also see Jaramillo and Steiner, 2014: 19).

## Investments and Divestments

What does it mean to invest in or divest from deliberative capital? Drawing on the deliberative democracy literature, I define investments as deliberative standards, norms or (pre)conditions, whereas divestments are solecisms that can occur during the process of deliberation. It is important to note that while we speak of *norms* or *solecisms* in the abstract, in real deliberative settings attention needs to be paid to other factors such as mannerisms, tone, context and even body language in order to identify investments and divestments. For example, interrupting someone’s speech can be a sign of *disrespect* in some circumstances or it may be an indicator of listening/reflection if, for instance, the other person is struggling to find a word and the interruption is an attempt to help a fellow interlocutor. Below, I offer an account of these investments, divestments and their respective indicators.^10^

### Investments

Investments refer to instances of adherence to particular deliberative standards or norms of justification (see, for example, Benhabib, 2002; Bohman, 1996; Fearon, 1998; Gutmann and Thompson, 2004; Habermas, 1996); respect (see Bohman and Richardson, 2009; Dryzek et al., 2011; Forester, 2009; Gastil, 2008, to name a few); listening, reflection on and incorporation of the views of others (see, for example, Chambers, 2009; Deveaux, 2006; Goodin, 2003; Habermas, 1984; He, 2010; Jungkunz, 2013; Karpowitz et al., 2009; Rawls, 1971); sincerity (Fishkin, 2011; Lenard, 2008; Warren, 2006); empathy (Mansbridge, 1980; Morrell, 2007, 2010); and productive dialogue – offering mediating proposals or accepting compromises (Steenbergen et al., 2003; also see O’Flynn and Setälä, 2020; Rostbøll, 2017; Weinstock, 2017).

Since these norms have been well established within the literature on deliberative democracy, I forgo providing a normative argument or an ethical rationale for why each serves as investments in deliberative capital. Instead, I draw on my experience as a facilitator to provide a guideline for the particular indicators of these investments – what we can look for as evidence that this particular investment has taken place. This can be seen in Table 1.^11^

**Table 1.** Indicators of Investments in Deliberative Capital.

Reason-giving
Justification
Explanation to make the meaning more intelligible
Respect
Absence of negative statements in expressing disagreement
Absence of interruptions in longer speech acts
Asking others what they think
Rephrasing/repeating what someone else has said
Apologizing for a negative deliberative behaviour
Using ‘we’ or ‘our’
Reflection and incorporation
Expressing change or amending of one’s view
Connect one’s point to general ideas
Connect one’s point to others’ ideas
Asking clarifying questions
Sincerity
Admittance of ignorance or lack of knowledge
Consistency in reasons given
Empathy
Identifying one’s own emotions
Acknowledging/communicating the feelings of others
Connecting one’s own feelings to others’ emotion (can be through an example)
Productive dialogue
Offering concessions
Offering mediating proposals
Separating personal feelings from positions

### Divestments

Divestments or negative deliberative behaviours refer to instances of disrespect, hermeneutical exclusion – or failing to extend arguments as they ‘go past each other by using incommensurate terms and meanings’ (Pearce and Littlejohn, 1997: 72); rhetorical action – or engaging in deliberation in order to simply ‘justify [one’s] own standpoint’ instead of engaging in a real back-and-forth with others (Bächtiger et al., 2010: 51);^12^ and unproductive dialogue – or instances where participants make no ‘attempt[s] at compromise, reconciliation, or consensus building’ (Steenbergen et al., 2003: 30); as well as moments where participants do not offer a justification for their positions; when they share or process information in a biased or incomplete manner (Myers, 2010, 2018); or when they engage in cognitive apartheid – or when they fail ‘to engage with one’s interlocutor as a person of intelligence’ by ‘[reconsidering] her views on basis of reasons’ (Bohman and Richardson, 2009: 270) and in doing so disrupt the flow of the discussion (Jaramillo and Steiner, 2014: 2). Table 2 details the indicators for each of them – what one needs to look for in a deliberation to see if negative deliberative behaviours are taking place.^13^

**Table 2.** Indicators of Divestments from Deliberative Capital.

Unsupported claims
No justification
No attempts to make a point more intelligible
Biased information sharing and processing
Pushing for false consensus
Presenting or being swayed by arguments evoking fear
Logical fallacy
Cognitive apartheid
Ignoring what others are saying – changing the flow drastically
Not taking into account any of the others’ real concern
Disrespect
Ad hominem attacks or hypocrisy
Cutting others off
Hermeneutical exclusion
Using the same term to mean different things
Misunderstandings without resolution
Rhetorical action
Dominating speech
Overconfidence in one’s view
Repetition of the same idea in the face of challenges
Silencing of speech acts opposed to one’s view
Unproductive dialogue
Rejection of mediating proposals
Rejection of concessions

## Incentivizing Investments and Discouraging Divestments

We know that divestments occur in deliberations. Are there ways that we can encourage investments and decrease the instances of divestments within deliberations? One key advantage of using the framework of deliberative capital is that it better accounts for the role of facilitators. This allows us to envision and devise strategies directed at incentivizing investments and discouraging divestments.

While the ‘myth of the neutral mediator’ (Pearce and Littlejohn, 1997, 21) tells us otherwise, facilitators are committed to ‘the generative quality of [. . .] deliberative conversations and mutually crafted agreements’ (Forester, 2009, 26). This means that while, like referees at a soccer match, they might be impartial towards the outcomes of a deliberation, they are concerned by the ‘means by which’ the deliberation is conducted (Pearce and Littlejohn, 1997, 105). In this section, I briefly outline and describe two strategies – facilitative techniques of simulated representation and deliberative worth exercises – aimed at increasing this ‘generative quality’ by incentivizing investments and discouraging divestments.

Facilitative techniques are mechanisms or exercises that can be utilized either at the start of the deliberation or during the course of the deliberation to change the conditions under which the deliberation takes place in order to facilitate participants’ investment in deliberative capital. ‘In structured deliberation[s], face-to-face disagreement is often mediated through moderators, facilitators, debating rules, civility codes, turn taking, shared information, and many other mechanisms designed to manage disagreement’ (Chambers, 2013: 205). However, simple mediation is often not enough to eradicate divestments arising from unequal power relationships. Facilitative techniques are tools in the box of strategies and innovative designed aimed at making deliberation more democratic and equal. Here, I introduce two such techniques: simulated representation and deliberative worth exercises.

### Simulated Representation

This facilitative treatment aims to get individuals in a particular deliberation to take steps to understand their fellow interlocutors – and the specific ways in which their different experiences, backgrounds and, even, their use of language differ from one another by presenting the positions of each other in a deliberation.

The exercise proceeds as follows: Participants will have a preliminary round of deliberation aimed at demonstrating the different positions at the table. After this round, participants are divided in groups of two or three and asked to carry out an in-depth interview with one another. In particular, the participants are asked to inquire after the positions of their fellow interlocutors and the reasons, feelings and motivations behind those positions. This interview round is succeeded with a round of deliberation where participants present and defend the positions of their group member(s).^14^ For instance, Person X is tasked with presenting and defending the positions of Person Y and vice versa. Once again, one can easily see how self-interest plays a role here: in order to ensure that your actual views and reasons are well represented, you are incentivized to do the same for your interview partner. After one round of deliberation with simulated representation in place, deliberation resumes in the normal way.

The impetus behind the facilitative treatment of simulated representation comes out of engagement with scholarship on both education and psychology. Pedagogy researchers have long highlighted the efficacy of role-playing exercises in the classroom as way to increase empathy of students towards one another as well to better learn and understand the sense of purpose and ‘the motivations of different historical, fictional, and imagined characters’ (Afsahi, 2020b: 7) for a brief overview of this scholarship, see Douglas and Coburn, 2009; Jarvis et al., 2002; Kodotchigova, 2002; Sumler-Edmond, 2013; Wender, 2014).

This pedagogical approach is vindicated in the research carried out by social and experimental psychologists. Time and time again, they have shown that both practices of imagined contact and perspective-taking are capable of altering or, rather, improving the attitudes and stances of individuals towards others, especially members of their *out-group* (see, for example, Galinsky et al., 2008; Galinsky and Moskowitz, 2000; Ku et al., 2010; Shih et al., 2009; Wang et al., 2014; West et al., 2015).

Finally, while deliberative democrats have long been aware of the positive impacts of *empathy exercises* in improving the positions of individuals towards more inclusive and empathetic ways (Morrell, 2007, 2010), recent research has demonstrated that perspective-taking exercises can indeed be used as a tool within deliberation (see Muradova, 2020).

This facilitative technique works in two parts. First, it can force us to move beyond simply defending our positions and values to teaching them to others. Teaching is a dialogical process and forces us to remain responsive to the other side to ensure that our efforts have been worthwhile. Second, it can force us to move simply beyond listening to others to really hearing them in order to articulate and defend them in the next round. Put simply, such a technique is designed to, hopefully, bring about a degree of ‘cognitive empathy’ in participants within a deliberation (Spencer, 1995).

Facilitative technique of simulated representation can be used to discourage divestments such as cognitive apartheid, rhetorical action, biased information processing and insufficient attempts at understanding or recognition as well as to encourage investments such as justification, listening to others, reflection, empathy and even attempts at a productive dialogue.

### Deliberative Worth Exercises

This facilitative treatment is aimed at addressing a key problem within deliberation: the possibility – if not the tendency – of participants forgetting about the norms and rules of deliberation. Deliberative worth exercises substitute the need to constantly remind participants of the norms of deliberation and instead push them to become cognizant of these norms and to internalize them more quickly.

The exercise proceeds as follows: Participants will be asked to pay attention to the behaviour of their fellow interlocutors especially their tendencies to invest in or divest from deliberative capital during each round of deliberation. At the end of each session of deliberation, participants are asked to think about who, in their opinion, was most successful at investing in deliberative capital and refraining from divestments and to write down this name on a piece of paper to preserve anonymity. The names are collected and read out at each round of deliberation. While this might seem to be a rather trivial practice, it operates by tapping into real cognitive, psychological and emotional needs of individuals.

The impetus behind deliberative worth exercises comes out engagement with the literature on sociology – especially scholarly work on ‘face-work’ and ‘face-saving’ strategies (Goffman, 1967, 1972, 2003; also see Bargiela-Chiappini, 2003; Brown and Garland, 1971; Lim and Bowers, 1991; Ting-Toomey, 2009). These scholars point to the drive by individuals to establish and preserve their image and reputation. As such, in social contexts with repeated interactions – such as a deliberation – individuals are and can be further motivated to maintain their face and reputation and ensure the continued favourable receptions from others.

Scholars within deliberative democracy have already highlighted the fact that the desire to maintain one’s reputation and self-image can act as a barrier to opinion change within deliberation (Sunstein, 2000, 2003). I argue this tendency can be used to benefit the process of deliberation. ‘Within a deliberative setting – especially one with explicit ground rules regarding the positive deliberative behaviors – participants can be encouraged to engage in face-saving strategies’ (Afsahi, 2020b: 7). Participants in a deliberation will be driven by their desire ‘for others to acknowledge their friendliness and honesty’ and to be viewed as ‘“likeable,” “acceptable,” “friendly,” “agreeable,” “cooperative,” “alike,” and “affiliated”’ (Huang, 2014: 180).

The facilitative technique of deliberative worth exercises can be utilized in order to discourage divestments such as insufficient attempts at respect, understanding or recognition; cognitive apartheid; hermeneutical exclusion; and rhetorical action as well as to encourage investments such as respect, empathy, listening to and incorporating the views of others, reflection and even attempts at a productive dialogue. The role of self-interest is perhaps even clearer in the case of this facilitative treatment. The self-interested impulse to be seen positively by others and to be acknowledged publicly as such leads participants to invest more readily, and as investments rise, deliberative capital increases and deliberation becomes better, more considered and more civil.^15^

## Conclusion

This article makes a number of important contributions to deliberative democracy. First, the concept of deliberative capital is useful at explaining the success and breakdown of speech and demonstrating how virtuous and vicious cycles are both equally possible in a deliberation. Second, in explaining the role that self-interest plays in incentivizing initial investments, the theory of deliberative capital offers a resolution to the problem of *pre-commitment* and defends deliberative democratic theory against accusations of excess idealism. While some interlocutors might be driven by their commitment to norms of deliberation and an attentiveness to common good, we do not need to hold out hope or cross our fingers for such *ideal* deliberators. In less ideal contexts, self-interest and a desire for reciprocity will deliver similar ends.

Finally, this framework better accounts for the role of facilitation within deliberation. As such, it puts forward two innovative facilitation methods that are tailored towards increasing investments and decreasing divestments within deliberation. Good deliberation happens when participants invest in deliberation and when skilful facilitation is able to stop instances of divestments turning into destructive cycles.

## Notes

1. I served as a facilitator for the Grandview Woodlands Citizens’ Assembly from August 2014 to May 2015. Furthermore, I served as a secondary moderator for the Forum on Best Practices for Child Protection Agency in March 2011. Finally, I acted as a key consultant for Innovative Research Group during the research and development phase of a series of deliberative mini-publics carried out by Toronto Hydro in June 2017. My particular role was to offer facilitation advice and guidelines to Innovative Research Group.

2. I first developed and presented the concept of deliberative capital in 2011 (Afsahi, 2011; also see Afsahi, 2014a, 2014b ) and received ethics approval for the project titled ‘Deliberative Capital’ in 2014 from the University of British Columbia, in Vancouver, Canada.

3. Human capital refers to the ‘knowledge, skills, and health’ of individuals (Becker, 1994: 16). It is increased through ‘[d]irect expenditures on education, [training,] health, and internal migration to take advantage of better job opportunities’ (Schultz, 1961: 1). Academic capital refers to ‘social processes that build family knowledge of educational and career options and support navigation through educational systems and professional organizations’ (John et al., 2010: 1). It is the ‘product of the combined effects of cultural transmission by the family and cultural transmission by the school’ (Bourdieu, 1984: 23). Cultural capital, meanwhile, ‘is the accumulation and knowledge, behaviors, and skills that one can tap into demonstrate one’s cultural competence, and thus one’s social status or standing in society’ (Cole, 2019). It manifests itself in an embodied form – ‘long-lasting dispositions of mind and body’; objectified form – ‘cultural goods [such as] pictures, book, [or] dictionaries’; as well as in an institutionalized form – ‘educational qualifications’ (Bourdieu, 1986: 47).

4. The only other utilization of the concept of deliberative capital is by Markus Holdo in a 2015 *Critical Policy Studies* article (Holdo, 2015). However, our uses of the concept are fundamentally different from one another. While both Holdo and I are concerned with deliberative democratic practices in organized structures (i.e. small-scale deliberative engagements), our approach to the concept is very different. For Holdo, deliberative capital is essentially different from competence, capacity and skill. It is the ‘source of social recognition’ particularly recognition outside the deliberative field as a result of the participation within it. However, I see deliberative capital as product of investments within deliberation. Deliberative capital is created and maintained when participants demonstrate themselves as capable – having the capacity – of making those investments. In addition, while I depend on the concept of deliberative capital to critically and analytically understand the moments of success and failure of speech within an organized dialogue, Holdo uses the concept to understand and explain the ways in which the voices of the marginalized, those without access to common forms of capital, can gain empowerment through engagement in a small-scale deliberative engagement. Broadly, while I examine investments in and divestments from deliberative capital in micro-deliberative settings, Holdo is interested in how small deliberative engagements can increase the deliberative capital of marginalized groups in macro-settings.

5. It is important to note that some scholars have pointed to the negative effects that social capital can have within societies as well (see, for example, Villalonga-Olives and Kawachi, 2017; Warren, 2004, 2008). Most notably, Alejandro Portes has highlighted four potential downsides of social capital. First, over-investment in social capital can lead to imposition of exorbitant demands on members. Second, social capital can operate as a form of informal social control by curtailing the freedom of individuals. Third, an excess of bonding social capital and a lack of bridging social capital can lead to the exclusion of out-group members. Finally, norm adherence as a result of reciprocity can lead to conformity (Portes, 1998; also see Arneil, 2006).

6. I should emphasize, however, as W. Barnett Pearce and Littlejohn (1997) do so eloquently in their work that I do not assume or suggest that ‘all participants in conflicts are well-meaning and moral people’. In fact, some might be ‘out to subvert others deliberately for personal gain, and such people can probably be found on side of most issues’ (Pearce and Littlejohn, 1997: 162). However, just as assuming that everyone is pre-committed to deliberative norms can undermine the process of deliberation, setting up, facilitating or participating in a deliberation with the assumption that everyone is morally corrupt will easily become a ‘self-fulfilling prophecy in which the other becomes as despicable as we treat them’ (p. 162).

7. I am not the first within deliberative democratic scholarship to highlight the place of self-interest within deliberation. Jane Mansbridge and her colleagues had previously discussed why pursuing your self-interest in a deliberation should not be seen as negative especially under conditions of unequal power relations where striving towards *common* interest might easily marginalize and silence individuals and groups lacking systemic power (Mansbridge et al., 2010). While I am sympathetic to this argument, my account of self-interest is strictly about the process of deliberation rather than about the substance of the issues discussed.

8. There is ample evidence for such a claim in the literature on game theory, especially the theories of *repeated games* (Jordan and Rand, 2017; Pacheco et al., 2008; Rand et al., 2009). Similarly, the literature on bargaining and negotiation also demonstrates a similar logic. In particular, Charles Osgood has shown that in most circumstances when participants cooperate – or invest – their cooperation is not exploited bur rather reciprocated (Osgood, 1962). In this case, ‘ [an] initial concession [sets] in motion a cycle of reciprocated or matched concessions’ (Druckman, 2011: 790).

9. The analysis by Stromer-Galley and her colleagues is guided by earlier research on disagreement within small-group settings (Pomerantz, 1984; Rendle-Short, 2007; see Kuo, 1994). Once again, the reciprocal nature of (poorly expressed) disagreement is not highlighted.

10. It has to be emphasized at this point that these investments and divestments – and their indicators – are associated with deliberation in small and structured settings *only*. Within the larger public sphere, some *investments* may work to further marginalize already-oppressed groups. For instance, insisting on an unqualified need for respect may undermine the ability of Black activists to draw attention to the daily violence experienced by members of their community, or it can hamstring our ability to call out far-right, White supremacist or fascist ideology in our politics.

11. A similar table can be seen in a previously published paper (Afsahi, 2020b: 12).

12. For more on rhetorical action, see Schimmelfennig (2001).

13. A similar table can be seen in a previously published paper (Afsahi, 2020b: 13).

14. I should perhaps emphasize here that under this facilitative technique, facilitators will be present and tasked with keeping the conversation on track and ensuring that basic deliberative standards are upheld.

15. I have previously examined and demonstrated the efficacy of simulated representation and deliberative worth exercises in increasing quality of deliberation and reducing the effects on power inequalities between men and women (see Afsahi, 2020b).
